# Ligand complex structures of l‐amino acid oxidase/monooxygenase from *Pseudomonas* sp. AIU 813 and its conformational change

**DOI:** 10.1002/2211-5463.12387

**Published:** 2018-02-08

**Authors:** Dohyun Im, Daisuke Matsui, Takatoshi Arakawa, Kimiyasu Isobe, Yasuhisa Asano, Shinya Fushinobu

**Affiliations:** ^1^ Department of Biotechnology The University of Tokyo Japan; ^2^ Department of Biotechnology Biotechnology Research Center Toyama Prefectural University Imizu Japan; ^3^ Asano Active Enzyme Molecule Project ERATO JST Imizu Japan; ^4^ Present address: Department of Cell Biology Graduate School of Medicine Kyoto University Yoshidakonoe‐cho, Sakyo‐ku Kyoto 606‐8501 Japan

**Keywords:** crystallography, flavin monooxygenases, flavin‐containing monoamine oxidase family, l‐amino acid oxidase/monooxygenase, l‐lysine, l‐ornithine

## Abstract

l‐Amino acid oxidase/monooxygenase from *Pseudomonas* sp. AIU 813 (l‐AAO/MOG) catalyzes both the oxidative deamination and oxidative decarboxylation of the α‐group of l‐Lys to produce a keto acid and amide, respectively. l‐AAO/MOG exhibits limited specificity for l‐amino acid substrates with a basic side chain. We previously determined its ligand‐free crystal structure and identified a key residue for maintaining the dual activities. Here, we determined the structures of l‐AAO/MOG complexed with l‐Lys, l‐ornithine, and l‐Arg and revealed its substrate recognition. Asp238 is located at the ceiling of a long hydrophobic pocket and forms a strong interaction with the terminal, positively charged group of the substrates. A mutational analysis on the D238A mutant indicated that the interaction is critical for substrate binding but not for catalytic control between the oxidase/monooxygenase activities. The catalytic activities of the D238E mutant unexpectedly increased, while the D238F mutant exhibited altered substrate specificity to long hydrophobic substrates. In the ligand‐free structure, there are two channels connecting the active site and solvent, and a short region located at the dimer interface is disordered. In the l‐Lys complex structure, a loop region is displaced to plug the channels. Moreover, the disordered region in the ligand‐free structure forms a short helix in the substrate complex structures and creates the second binding site for the substrate. It is assumed that the amino acid substrate enters the active site of l‐AAO/MOG through this route.

**Database:**

The atomic coordinates and structure factors (codes 5YB6, 5YB7, and 5YB8) have been deposited in the Protein Data Bank (http://wwpdb.org/).

**EC numbers:**

1.4.3.2 (l‐amino acid oxidase), 1.13.12.2 (lysine 2‐monooxygenase).

**Abbreviations**
l‐AAO/MOG: l‐amino acid oxidase/monooxygenase from *Pseudomonas* sp. AIU 813
LGOX: l‐Glu oxidase from *Streptomyces* sp. X‐119‐6
MAO: monoamine oxidase
PAO: l‐Phe oxidase (deaminating and decarboxylating) from *Pseudomonas* sp. P‐501
TMO: l‐Trp 2‐monooxygenase from *Pseudomonas savastanoi*

l‐Amino acid oxidase (EC 1.4.3.2, l‐AAO) catalyzes the oxidative deamination of the α‐group of l‐amino acid to produce an α‐keto acid, while l‐amino acid 2‐monooxygenase (EC 1.13.12.‐) catalyzes the oxidative decarboxylation to produce an amide. l‐Amino acid oxidase/monooxygenase from *Pseudomonas* sp. AIU 813 (l‐AAO/MOG) is a bifunctional enzyme that exhibits both the l‐Lys α‐oxidase and l‐Lys 2‐monooxygenase (EC 1.13.12.2) activities (Fig. 1) 1, 2. Most l‐AAOs show a wide range of substrate specificity, as represented by snake venom l‐AAOs 3. However, crystal structures of three l‐AAOs (and l‐amino acid 2‐monooxygenases) with strict substrate specificity have been reported: l‐Glu oxidase from *Streptomyces* sp. X‐119‐6 (LGOX) 4, l‐Phe oxidase (deaminating and decarboxylating) from *Pseudomonas* sp. P‐501 (PAO) 5, 6, and l‐Trp 2‐monooxygenase from *Pseudomonas savastanoi* (TMO) 7. The l‐AAOs are FAD‐dependent enzymes and members of the flavin‐containing monoamine oxidase (MAO) family 8. l‐AAO/MOG is classified into a distinct phylogenetic branch from other MAO family enzymes along with PAO and TMO 2. However, l‐AAO/MOG shows low or barely detectable amino acid sequence identity to these enzymes: 16.7% to LGOX, 18.8% to PAO, and 30.1% to TMO by EMBOSS Needle pairwise alignment. l‐AAO/MOG shows limited substrate specificity toward l‐amino acids with a positively charged side chain group. Relative oxidase activities of l‐AAO/MOG toward l‐ornithine (Orn) and l‐Arg compared with l‐Lys were 31% and 6%, respectively 1. In our previous study 2, a ligand‐free crystal structure of l‐AAO/MOG and its interconversion between oxidase and monooxygenase activities by chemical modification and point mutation were described. The key residue for the activity conversion (Cys254) is located behind the aromatic substrate‐binding cage but not directly involved in the interaction with the substrate.

**Figure 1:**
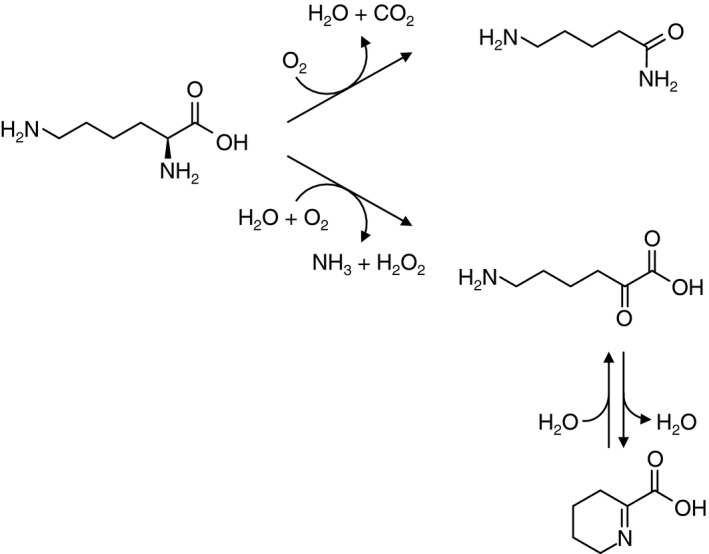
Reaction scheme of oxidative deamination (oxidase, lower path) and oxidative decarboxylation (monooxygenase, upper path) of l‐Lys by l‐ AAO/MOG.

In this study, we determined the crystal structure of l‐AAO/MOG complexed with l‐Lys, l‐Orn, and l‐Arg. Moreover, catalytic and kinetic features of mutant enzymes at a residue responsible for binding of the ε‐amino group were investigated. The structure of l‐AAO/MOG complexed with its substrates is compared with the ligand‐free structure, and conformational changes and entrances for the substrates through channels connecting the active site and solvent are discussed.

## Results

### Ligand complex structures

**Figure 2:**
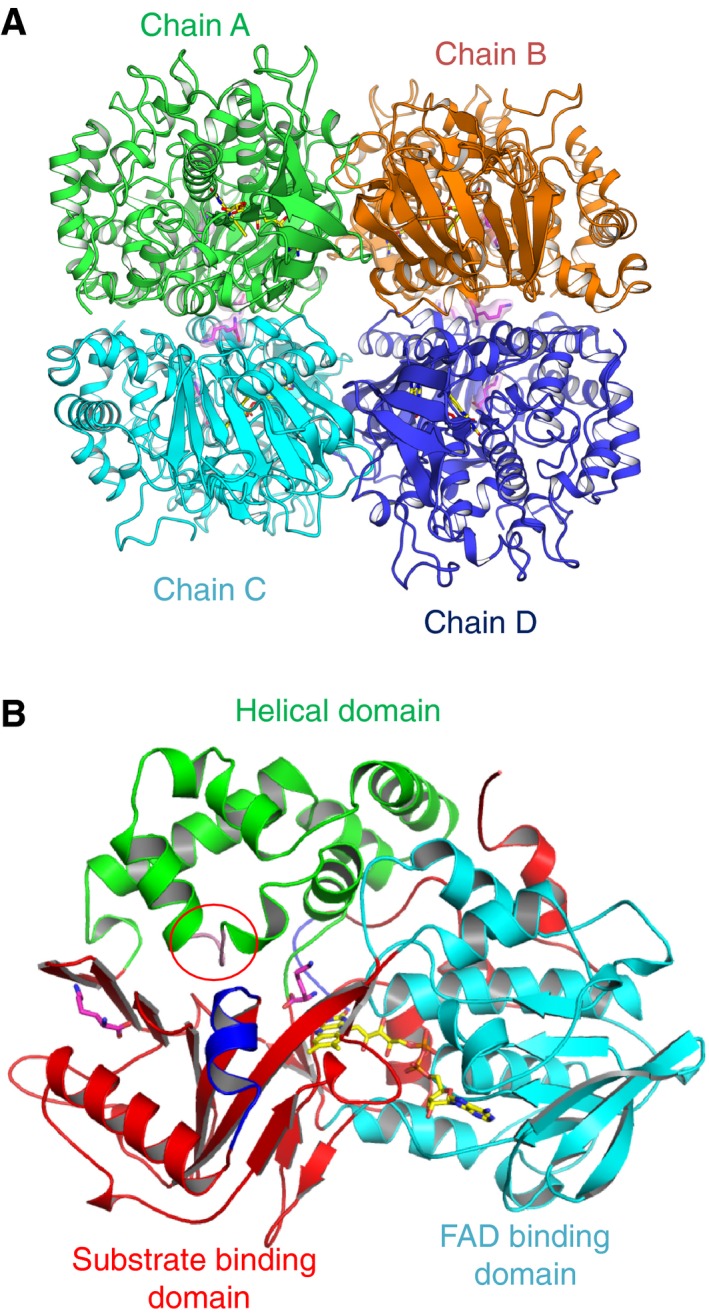
Overall structure of l‐ AAO/MOG. (A) Tetramer structure contained in the asymmetric unit. FAD and l‐Lys molecules are shown as yellow and magenta sticks. The biological dimers consist of chains A–C and B–D. (B) Monomer structure of l‐ AAO/MOG. The plug loop (in the helical domain, red circle) and flexible helix (in the substrate binding domain) are shown in pink and blue, respectively.

The structures of l‐AAO/MOG complexed with either l‐Lys, l‐Orn, or l‐Arg were determined at 2.0–2.3 Å resolution (Table 1). When yellow l‐AAO/MOG crystals were soaked in the crystallization buffer containing the substrate (l‐Lys, l‐Orn, or l‐Arg), the color changed to pale yellow, suggesting that the flavin cofactor was partially reduced. The X‐ray dose during the data collection was not extremely high (1.17 MGy as estimated by the Raddose‐3D server) 9, but it might have partially caused photoreduction of the crystal. However, conformations of the isoalloxazine ring in the complex structures showed no significant difference with that in the previously determined ligand‐free structure (PDB ID: 3WE0), indicating that the flavin in the crystal was not fully reduced and may contain mixed redox states. Because the ligand complex crystals were prepared under an aerobic condition, the substrate was possibly oxidized. However, we could not confidently determine the redox states of the flavin and the substrate (oxidized or unreacted). In the present crystal structure, we modeled an unreacted substrate in the active site, according to the previous crystallographic studies on l‐AAOs 6, 10, 11. The crystal structures of the complexes contain a tetramer in the asymmetric unit (Fig. 2A). It has been shown that l‐AAO/MOG forms dimer in solution 1; thus, the crystals contain two biological dimers in the asymmetric unit. The four monomers in the asymmetric units are virtually the same, and the RMSD for the Cα atoms between the chains is typically < 0.1 Å. Here, we mainly describe chain A and its biological dimer counterpart (chain C). The Cα RMSD between the complex structures (l‐Lys, l‐Orn, and l‐Arg) and the ligand‐free structure are ~ 0.75, ~ 0.55, and ~ 0.40 Å, respectively, indicating that the overall structure is not largely changed upon substrate binding. However, two short regions exhibit a significant structural change and/or lose flexibility upon ligand binding (described below). The monomer structure of l‐AAO/MOG (Fig. 2B) consists of three domains: FAD binding domain (blue), substrate binding domain (red), and helical domain (green).

**Table 1:** Data collection and refinement statistics

	l‐Lys complex	l‐Orn complex	l‐Arg complex
Data collection			
PDB entry	5YB6	5YB7	5YB8
Space group	*P*2_1_	*P*2_1_	*P*2_1_
Unit cell (Å)	*a* = 97.1, *b* = 132.3	*a* = 94.6, *b* = 132.9	*a* = 98.2, *b* = 133.0
	*c* = 101.0	*c* = 101.9	*c* = 101.5
	β (°) = 108.7	β (°) = 111.5	β (°) = 112.0
Resolution (Å)	50.00–2.10 (2.14–2.10)	94.81–2.00 (2.15–2.00)	94.11–2.30 (2.42–2.30)
Total reflections	473 819	590 825	404 779
Unique reflections	139 347	156 339	106 954
Completeness (%)	99.5 (99.7)	98.9 (98.0)	99.7 (99.8)
Redundancy	3.4 (3.3)	3.8 (3.7)	3.8 (3.8)
Mean *I*/σ	10.6 (2.9)	16.0 (3.7)	12.0 (3.2)
*R*_merge_ (%)	12.6 (35.1)	5.4 (40.7)	8.1 (47.2)
Refinement			
Resolution (Å)	50.00–2.10	94.81–2.00	94.11–2.30
No. of reflections	132 300	144 806	104 920
*R*/*R*_free_ (%)	23.7/28.3	19.8/24.0	22.0/27.3
No. of atoms	18 833	18 307	17 837
No. of solvents	8 (Lys), 4 (FAD), 4 (PEG)	8 (Orn), 4 (FAD)	8 (Arg), 4 (FAD)
RMSD from ideal values			
Bond lengths (Å)	0.017	0.017	0.015
Bond angles (°)	1.80	1.77	1.73
Ramachandran plot (%)			
Favored	96.1	96.8	97.2
Allowed	3.3	2.7	2.2
Outlier	0.59	0.55	0.59

Values in parentheses correspond to the highest resolution shell.

**Figure 3:**
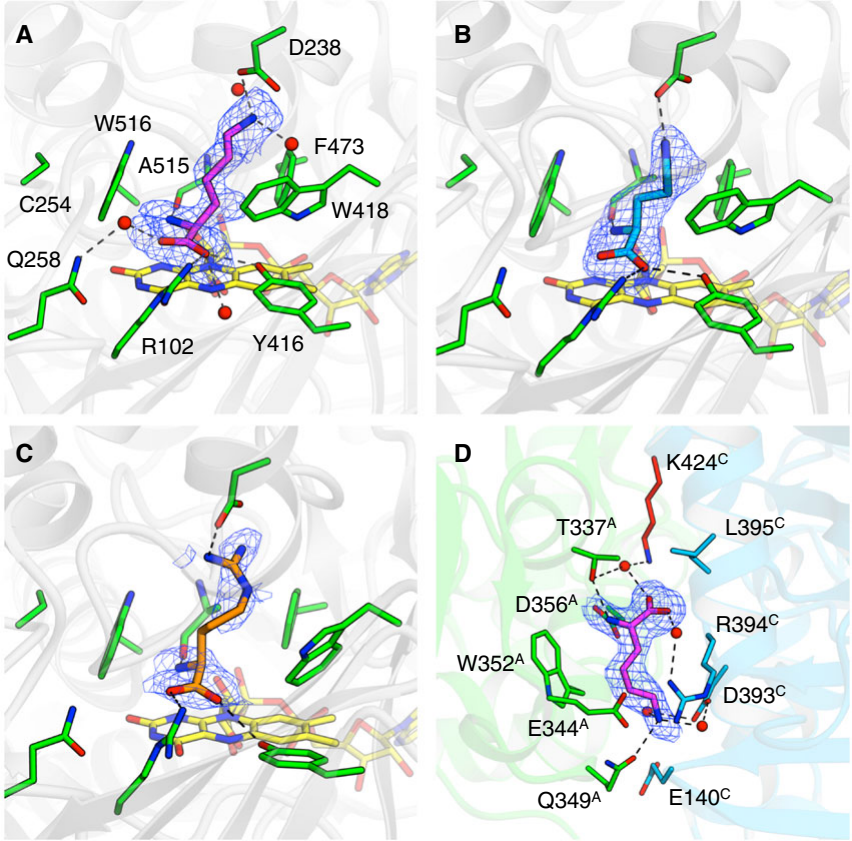
Substrate binding sites of l‐ AAO/MOG. (A) l‐Lys (magenta), (B) l‐Orn (cyan), and (C) l‐Arg (orange) in the active site. FAD molecule is shown as yellow sticks. (D) The second substrate binding site between the dimer of chains A and C. *mF*_o_–*F*_c_ omit electron density maps (2.5σ, blue mesh) are shown.

The active site of the complex structures is shown in Fig. 3A–C. The electron density map for a l‐Lys molecule was clearly observed in the substrate binding pocket (Fig. 3A). In the active site of MAO family enzymes, an ‘aromatic cage’ formed by the re‐side of the isoalloxazine ring of FAD and two aromatic residues is generally present 12. Phe473 and Trp516 in l‐AAO/MOG correspond to the two aromatic residues of the ‘cage’. In addition to these residues, the butylene (‐C_4_H_8_‐) group of the bound l‐Lys is surrounded by Trp235 (not shown in Fig. 3 for clarity), Trp418, and Ala515. The α‐amino and carboxyl groups are recognized by ionic interactions and direct hydrogen bonds from the side chains of Arg102 and Tyr416 and the main chain carbonyl group of Ala515, as well as water‐mediated hydrogen bonds from the side chain of Gln258. The ε‐amino group directly interacts with the side chain of Asp238. This acidic residue forms a ceiling of a long hydrophobic pocket and is considered to be responsible for the substrate selectivity. The electron density maps for l‐Orn and l‐Arg were relatively ambiguous (Fig. 3B,C). The positively charged side chain moiety of l‐Arg showed very weak electron density at its midchain area. However, these substrates also retain the charge‐based interaction with Asp238. Furthermore, clear electron density of a l‐Lys molecule was observed in the dimer interface (Fig. 3D). Residues from both monomers, including Lys424 in the flexible helix, are involved in the recognition. Glu140, Glu344*, Gln349*, and Asp393 (asterisks indicate that these residues are from the neighboring monomer) are involved in the recognition of the ε‐amino group of the bound l‐Lys. Because a similarly bound ligand was also observed in l‐Orn and l‐Arg complex structures (data not shown), this is the second binding site for the basic l‐amino acid substrates.

**Figure 4:**
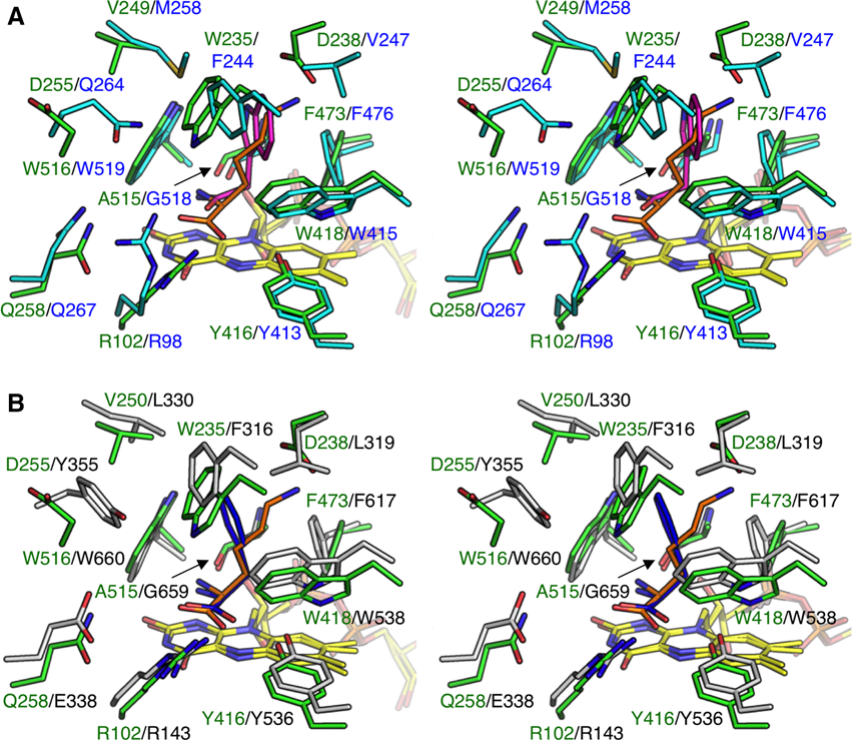
Comparison of the active site with other l‐ AAOs (stereoviews). The structure of the l‐ AAO/MOG (green) complex with l‐Lys (orange) is superimposed with (A) l‐Trp 2‐monooxygenase (TMO) from *Pseudomonas savastanoi* (cyan) in complex with 2‐indoleacetamide (magenta, PDB ID: 4IV9) and (B) l‐Phe oxidase (PAO) from *Pseudomonas* sp. P‐501 (gray) in complex with l‐Phe (blue, PDB ID 3AYJ). The two structures were superimposed using FAD molecule.

The active site in the structure of l‐AAO/MOG complexed with l‐Lys was superimposed with those of other l‐AAOs specific for l‐Trp (TMO, Fig. 4A) 7 and l‐Phe (PAO, Fig. 4B) 6. The conformations of FAD, the aromatic cage residues, and residues involved in the recognition of α‐amino and carboxyl groups are basically conserved. Corresponding to the specificities of TMO and PAO for larger aromatic l‐amino acids, their pockets are wider than that of l‐AAO/MOG through substitutions of Trp235 to Phe244/316 and Ala515 to Gly518/659. The corresponding residues of Asp238 are Val247 and Leu319 in TMO and PAO, respectively, suggesting that this is a key residue for the amino acid substrate specificity of l‐AAOs.

### Mutational analysis

To investigate the functional role of Asp238, we initially constructed three site‐directed mutant proteins: D238A (short side chain), D238K (basic residue), and D238E (acidic residue). These mutant proteins exhibited similar spectral features to the wild‐type enzyme (Fig. S1A). Although D238K lost activity, the l‐Lys oxidase activity of D238A and D238E exhibited classical Michaelis–Menten kinetics similar to the wild‐type enzyme (Fig. S1B). We determined the kinetic parameters of the wild‐type and D238A and D238E mutant enzymes toward l‐Lys, l‐Orn, and l‐Arg by separately measuring the oxidase and monooxygenase activities (Table 2). The D238A mutant showed increased *K*_m_ values for all of the three substrates, and the *k*_cat_ values for l‐Lys and l‐Orn decreased. The *k*_cat_/*K*_m_ values for all of the three substrates significantly decreased. Therefore, removal of the carboxyl group at this position impaired the enzymatic function for the basic substrates. Substitution to Lys (D238K) reduced the activity to an undetectable level because charge repulsion was introduced at this site. Interestingly, the D238E mutant exhibited higher activity (increased *k*_cat_ and decreased *K*_m_) toward l‐Lys compared with the wild‐type, but the activities toward l‐Orn and l‐Arg decreased. Elongation of the side chain by one methyl group may fine‐tune the interaction between the carbonyl (Glu238) and the ε‐amino (l‐Lys substrate) groups. In Table 2, the oxidase/monooxygenase activity ratios based on the *k*_cat_ values are shown. The mutant enzymes did not show significant changes on the oxidase/monooxygenase activity ratio toward l‐Lys.

**Table 2:** Kinetic parameters of wild‐type and mutant enzymes of l‐AAO/MOG

Enzyme^a^	Substrate	Oxidase			Monooxygenase			O/M ratio^b^
		*k*_cat_ (s^−1^)	*K*_m_ (mm)	*k*_cat_/*K*_m_ (s^−1^·mm ^−1^)	*k*_cat_ (s^−1^)	*K*_m_ (mm)	*k*_cat_/*K*_m_ (s^−1^·mm ^−1^)	
Wild‐type	l‐Lys	0.80 ± 0.09	0.027 ± 0.003	30 ± 1	4.5 ± 0.1	0.061 ± 0.006	74 ± 5	0.18
	l‐Orn	1.5 ± 0.2	0.022 ± 0.004	68 ± 1.8	6.7 ± 0.6	0.033 ± 0.001	200 ± 10	0.22
	l‐Arg	0.41 ± 0.02	0.048 ± 0.001	8.5 ± 0.65	1.6 ± 0.2	0.067 ± 0.005	24 ± 1	0.26
D238A	l‐Lys	0.50 ± 0.03	0.61 ± 0.05	0.82 ± 0.09	2.7 ± 0.4	0.26 ± 0.02	10 ± 1	0.19
	l‐Orn	0.37 ± 0.05	0.45 ± 0.02	0.82 ± 0.10	0.29 ± 0.01	0.31 ± 0.01	0.94 ± 0.04	1.3
	l‐Arg	0.10 ± 0.02	0.41 ± 0.01	0.24 ± 0.06	2.8 ± 0.4	0.42 ± 0.04	6.7 ± 0.3	0.036
D238E	l‐Lys	2.3 ± 0.3	0.018 ± 0.006	130 ± 10	8.1 ± 0.6	0.043 ± 0.001	190 ± 10	0.28
	l‐Orn	0.58 ± 0.03	0.041 ± 0.004	14 ± 0.3	2.3 ± 0.2	0.061 ± 0.004	32 ± 2	0.25
D238F	l‐Lys	0.17 ± 0.01	0.049 ± 0.009	3.5 ± 0.2	0.80 ± 0.04	0.072 ± 0.003	11 ± 1	0.21
	l‐Arg	0.10 ± 0.01	0.080 ± 0.018	1.3 ± 0.1	1.2 ± 0.29	0.090 ± 0.004	13 ± 1	0.083

Reaction at 30 °C in 100 mm potassium phosphate (pH 7.0 for l‐Lys as a substrate) and borate–NaOH (pH 9.0 for l‐Arg and l‐Orn as substrates) using 1.0 μm l‐AAO/MOG (final concentration). For *k*_cat_ and *K*_m_, means and 95% confidence limits of replicate assays are shown. ^a^ Activities of D238K, D238V, and D238N were not detected. The kinetic parameters of D238E for l‐Arg and D238F for l‐Orn were not determined because of undetectable activity. ^b^ Ratios of the oxidase/monooxygenase activities based on the *k*_cat_ values.

These two mutant enzymes exhibited no activity on amino acids other than l‐Lys, l‐Orn, and l‐Arg. To further explore the effects of substitution at Asp238, we performed random mutagenesis at this site. To find a mutant protein with altered substrate specificity, the mutant library was screened by an oxidase activity assay (detection of H_2_O_2_) using a mixture of l‐amino acids except for l‐Lys, l‐Arg, and l‐Orn as substrates. By the screening method, we could only obtain one mutant enzyme, D238F. The oxidase activities of the wild‐type enzyme and D238F toward various amino acids were measured (Table 3). The activity of D238F on l‐Lys decreased by approximately fivefold compared with the wild‐type enzyme, and the activity on l‐Orn was almost undetectable. The kinetic parameters for l‐Lys indicated that the mutation largely reduced the *k*_cat_ value, and there was no significant oxidase/monooxygenase activity conversion (Table 2). D238F retained the activity toward l‐Arg, probably due to the partially hydrophobic nature of the arginine side chain. D238F exhibited extended substrate specificity as it was also active on l‐Ala (Table 3). Interestingly, the mutant enzyme exhibited a significant activity toward decarboxylated compound of lysine, cadaverine. However, it is unclear why the Asp to Phe mutation made the enzyme active on the diamine compound. As expected by the method of screening and the large hydrophobic side chain of Phe residue, D238F demonstrated significant catalytic activity on amino acids with a long hydrophobic side chain, l‐Leu, l‐Met, and l‐Phe. The activities of D238F toward these hydrophobic amino acids were higher than the l‐Lys oxidase activity at high substrate concentration (5 mm). Therefore, we could modify the substrate specificity of l‐AAO/MOG by mutating Asp238, indicating that this residue plays a critical role in substrate recognition. From the random mutagenesis library, we also obtained D238V and D238N mutants, but they showed no activity on all substrates we tested (data not shown).

**Table 3:** Substrate specificity of wild‐type and D238F mutant enzymes of l‐AAO/MOG

Substrate^a^	Wild‐type		D238F	
	0.1 mm	5 mm	0.1 mm	5 mm
l‐Lys	0.26 ± 0.05	0.53 ± 0.04	0.059 ± 0.01	0.13 ± 0.01
l‐Orn	0.28 ± 0.01	0.13 ± 0.02	0.009 ± 0.0002	0.0025 ± 0.0001
l‐Arg	0.11 ± 0.008	0.11 ± 0.01	0.011 ± 0.001	0.11 ± 0.01
l‐Ala	−b	−b	0.012 ± 0.0005	0.0085 ± 0.0005
l‐Leu	−b	−b	0.024 ± 0.003	0.38 ± 0.03
l‐Phe	−b	−b	0.010 ± 0.002	0.055 ± 0.005
l‐Met	−b	−b	0.011 ± 0.008	0.23 ± 0.01
Cadaverine	−b	−b	0.021 ± 0.001	0.0119 ± 0.0004

Oxidase activity (units·mg^−1^) against 23 amino acid substrates (0.1 or 5 mm) was measured (*n* = 3) at pH 7.0 and 30 °C as described in Materials and methods. ^a^ Both enzymes showed no activity to l‐His, l‐Ser, l‐Thr, l‐Asn, l‐Gln, l‐Asp, l‐Glu, Gly, l‐Val, l‐Ile, l‐Tyr, l‐Trp, l‐Cys, l‐Pro, d‐Lys, d‐Orn, and d‐Arg. ^b^ Not detected.

## Discussion

### Channels into the pocket

**Figure 5:**
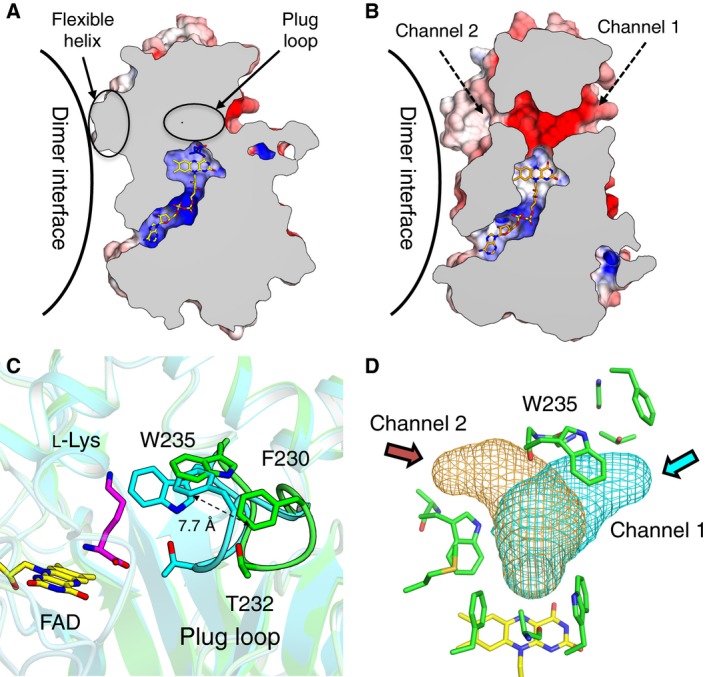
Conformational change between the ligand‐free and l‐Lys complex structures of l‐ AAO/MOG. Cross section of a monomer of (A) l‐Lys complex and (B) ligand‐free structures showing the internal cavities and channels. (C) Active site of ligand‐free (green) and l‐Lys complex (cyan). (D) Channels to solvent are displayed as meshes.

**Figure 6:**
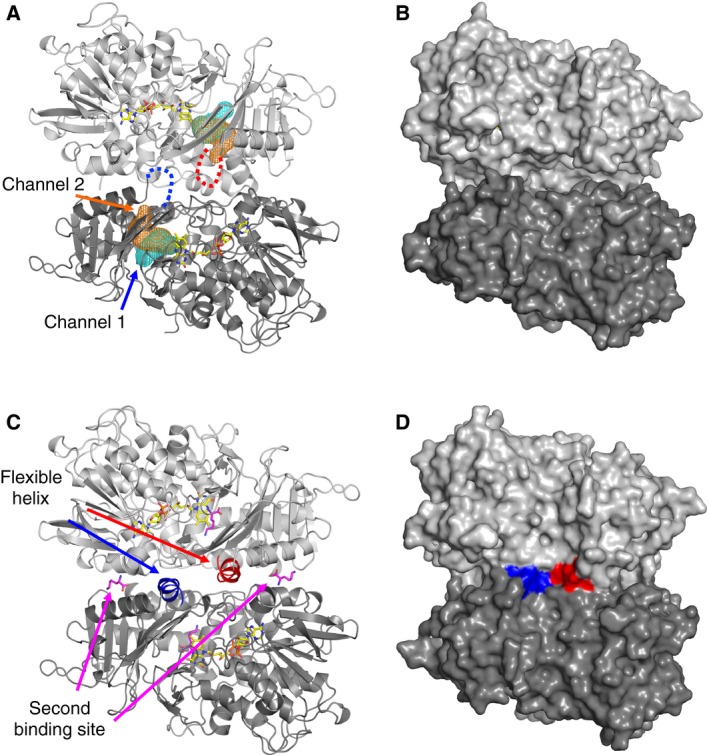
Dimer structures of (A, B) ligand‐free and (C, D) l‐Lys complexed with l‐ AAO/MOG shown by (A, C) cartoon and (B, D) surface representations. Flexible helices at the dimer interface are shown as blue and red. Dotted lines in (A) indicate that this region is disordered in the ligand‐free structure. Channel 1 (cyan) and channel 2 (orange) are shown as mesh in (A). l‐Lys and FAD molecules are shown as magenta and yellow sticks, respectively, in (A and C).

In the structure of l‐AAO/MOG complexed with l‐Lys, the substrate binding pocket of l‐AAO/MOG is completely occluded from solvent (Fig. 5A). In contrast, two channels connecting the active site and solvent are present in the ligand‐free structure (Fig. 5B). Channel 1 faces outward toward the dimer assembly, whereas channel 2 faces the dimer interface. The channels are located between the substrate binding and helical domains (Fig. 2B). The structural change mostly occurs in a loop region (Val228‐Trp235) with a maximum shift of 7.7 Å at the side chain atom of Phe230; thus, we named it the ‘plug loop’ (Fig. 5C). The plug loop consists of four glycines and mostly hydrophobic residues with the amino acid sequence VGFGTGGW, suggesting that it is intrinsically flexible. Trp235, which forms a hydrophobic wall of the pocket for l‐Lys, displaces approximately 5.0 Å, and this movement is mainly responsible for opening/closing channel 1 (Fig. 5D). Therefore, we assume that the two channels are entrance routes for the substrates. Presence of the second binding site near the entrance of channel 2 suggests that the l‐amino acid substrate may access through this route (Fig. 6). Notably, the occluded substrate pocket by the closed state of the plug loop was only observed in the l‐Lys complex structure, not in the l‐Orn and l‐Arg complex structures. The lower activities toward the latter two substrates may be due to the insufficient closing of the active site. On the other hand, channel 1 may be a route for oxygen because the entrance of this tunnel is present on the molecular surface (Fig. 6A). Tunnel‐like substrate access channels have been also found in other amino acid oxidases and monooxygenases. Ida *et al*. 5, 6 identified two tunnels in the l‐Phe complex structure of PAO and proposed that they are channels for the substrate and oxygen. However, the two channels, namely ‘oxygen channel’ and ‘substrate channel’, in PAO are connected to the molecular surface through different routes from those of the channels 1 and 2 in l‐AAO/MOG (data not shown). In the case of TMO, a large active site cavity containing FAD and indole‐3‐acetamide (reaction product) has three openings to the molecular surface 7. They provide access to the adenosine and the pyrophosphate groups of FAD, and the third tunnel was suggested to be a channel for the substrate.

### Dimer structure and possible substrate approaching routes

In the ligand‐free structure, a region connecting β16 and α15 (Met419–His428) was not included in the final model due to disorder (Fig. 6A, red and blue dotted lines) 2. In the three complex structures determined in this study, this region was clearly observed as a 2‐turn α‐helix (Fig. 6C, red and blue helices). Therefore, we named this region the ‘flexible helix’. The flexible helix is located near the dimer interface and the entrance of channel 2. Molecular surfaces of the ligand‐free (Fig. 6B) and l‐Lys complex (Fig. 6D) structures illustrate that the flexible helix acts as a lid of channel 2 because fixing of this region fills in the entrance hole at the dimer interface. Presence of the second binding site near the flexible helix (Fig. 6C) strongly suggests that the amino acid substrate enters through this route (channel 2). Although we have no evidence for the entrance of the other substrate (oxygen), the presence of channel 1 on the outer surface indicates that oxygen can easily access from solvent through this channel. In the case of flavin‐dependent *p*‐hydroxyphenylacetate hydroxylase and alditol oxidase, an extensive study using X‐ray crystallography, molecular dynamics simulations, rapid kinetic experiments, and site‐directed mutagenesis indicated that there are multiple diffusion pathways for oxygen into the active site 13.

Observation of the open–close states at the two channels by the conformational changes of the plug loop and flexible helix may indicate a possible control mechanism for substrate capture and product release by l‐AAO/MOG because the amino acid substrate and oxygen are required at different reaction steps in the catalytic cycle. The catalysis of flavin‐dependent oxidase/monooxygenases generally consists of two half‐reactions by which the flavin is alternately reduced and oxidized 14. In the reductive first half‐reaction, in which a hydride transfer most likely occurs in l‐AAOs (dehydrogenation), the amino acid substrate must enter the active site to place its Cα atom near the N(5) atom of the isoalloxazine ring 11. For the oxidase reaction, the oxidized imino acid intermediate is hydrolyzed to produce α‐keto acid and NH_4_^+^ (deamination) 6. In the oxidative half‐reaction, the flavin is re‐oxidized by dioxygen (electron acceptor), and H_2_O_2_ is produced from the oxidase reaction. For monooxygenases such as *p*‐hydroxybenzoate hydroxylase 15 and *p*‐hydroxyphenylacetate hydroxylase 13, a C4a‐hydroperoxide adduct has been observed during the oxidative half‐reaction of the reduced flavin with molecular oxygen. As for flavin‐dependent oxidases, the C4a‐hydroperoxide adduct has been also observed in pyranose 2‐oxidase 16. Ida *et al*. 6 proposed that the C4a‐hydroperoxide intermediate is formed at this step of the monooxygenase reaction of PAO, and an oxygen atom is subsequently inserted into the substrate and produces amide via decarboxylation and dehydration. However, it is not known if the similar C4a‐hydroperoxide intermediate is formed in the monooxygenase reaction of l‐AAO/MOG.

Our previous study demonstrated that chemical modification and mutation at Cys254, which is located at the ‘back’ side of Trp516, significantly changes the oxidase/monooxygenase activity ratio 2. In particular, the oxidase activity of C254I mutant enzyme increased by fivefold than that of the wild‐type enzyme, whereas its monooxygenase activity decreased to undetectable level. In the complex structure with l‐Lys, the α‐amino group points to the aromatic side chain of Trp516 (Fig. 3A), suggesting that correct binding of the Cα‐N (amino or imino) group on the isoalloxazine ring is required for the oxygen insertion step from the hydroperoxide group in the monooxygenase reaction. The oxidase/monooxygenase activity ratio was within a range of 0.18–0.28 for the wild‐type enzyme and D238E mutant (Table 2), suggesting that the mutation preserving the carboxyl side chain did not affect the reaction mechanism toward all substrates. The ratio toward non‐l‐Lys substrates was large (1.3 for D238A toward l‐Orn) or small (< 0.1 for D238A and D238F toward l‐Arg) in the cases of the substitutions with noncharged residues. The D238A and D238F mutations may also change the binding position for these substrates.

## Conclusion

Previously, we determined the crystal structure of l‐Lys ε‐oxidase (LodA) from *Marinomonas mediterranea* 17. However, in contrast to common amino acid oxidases that act on the α‐amino group, LodA is a cysteine tryptophylquinone‐dependent enzyme and employs a completely different system for the recognition and catalysis of the oxidase reaction of the side chain ε‐amino group.

The substrate recognition and reaction mechanisms of LGOX (l‐Glu oxidase) 4, PAO (l‐Phe/l‐Tyr oxidase/monooxygenase) 6, and TMO (l‐Trp 2‐monooxygenase) 7 have been studied by their ligand complex crystal structures. In the case of l‐Lys α‐oxidase from *Trichoderma viride* (sequence identity = 18.8%), a possible binding mode of l‐Lys to the enzyme was estimated by modeling the substrate in the active site of the ligand‐free crystal structure 18. In this study, we revealed the detailed structural basis for the recognition of positively charged amino acid substrates by determining the substrate complex structures of l‐AAO/MOG, and mutational analysis demonstrated that Asp238 plays a critical role in the substrate specificity. The D238F mutant exhibited altered specificity for long hydrophobic substrates (l‐Leu, l‐Met, and l‐Phe). The oxidase activity of the mutant enzyme can be used for spectrophotometric determination of those compounds by detecting the produced H_2_O_2_ by 4‐aminoantipyrine and an additional chromogen (e.g., phenol or *N*,*N*‐diethylaniline) 19. Colorimetric determination of amino acids can be used in diagnosis and screening of inborn diseases with metabolism errors 20, 21, 22.

## Materials and methods

### Materials

l‐Lys, l‐Orn, l‐Arg, and other amino acids were purchased from Wako Pure Chemical Industries (Osaka, Japan). Restriction endonucleases and the ligation reaction mixture were obtained from Toyobo (Osaka, Japan) and Takara Bio (Shiga, Japan). All other chemicals were purchased from Kanto Kagaku Co. (Tokyo, Japan), Nacalai Tesque Inc. (Kyoto, Japan), Sigma‐Aldrich Co. (St. Louis, MO, USA), or Tokyo Kasei Kogyo (Tokyo, Japan), unless otherwise stated, and were of the highest commercially available grade.

### Protein preparation and crystallography

N‐terminally His‐tagged recombinant l‐AAO/MOG protein was expressed in *Escherichia coli* and purified as described previously 2. The purified protein was dialyzed against 20 mm HEPES–NaOH (pH 7.0) and concentrated using Amicon centrifugal filters (Millipore, Billerica, MA, USA) with a 30 kDa cutoff membrane to 20 mg·mL^−1^ for crystallization. Protein crystallization was performed by sitting‐drop vapor diffusion method at 20 °C by mixing 1 μL of protein solution and 1 μL of reservoir solution. The reproducibility of the crystallization conditions described in the previous report (8% PEG4000 and 0.1 m sodium acetate, pH 4.6) was not high enough for extensive soaking experiments. After crystallization screening from scratch, a new condition with high reproducibility was found. Native l‐AAO/MOG crystals were obtained using reservoir solution consisting of 20% of PEG3350 and 0.15 m dl‐malic acid, pH 7.0. Complex crystals were prepared by soaking the crystals for 10–30 s in a reservoir solution supplemented with 20% glycerol and 10 mm l‐Lys, 20 mm l‐Orn, or 20 mm l‐Arg. Crystals were flash‐cooled at 100 K in a stream of nitrogen gas. Diffraction data were collected at beamline BL5A at the Photon Factory, High Energy Accelerator Research Organization (KEK; Tsukuba, Japan) at a wavelength of 1.000 Å. The diffraction images were processed using hkl2000 (l‐Lys complex) 23 and xds (l‐Orn and l‐Arg complex) 24. The initial phases of the ligand complex structures were solved by molecular replacement using the native l‐AAO/MOG structure (PDB ID: 3WE0) as a search model. Manual model rebuilding and refinement were achieved using coot 25, refmac5 26, and phenix 27. The resultant *F*_o_–*F*_c_ and 2*F*_o_–*F*_c_ maps yielded an electron density that corresponded to the soaked substrate. Figures were prepared using Open Source pymol (Schrödinger, LLC, New York, NY, USA), cuemol (http://www.cuemol.org/), and caver 3.0 28.

### Mutagenesis

Mutations were introduced by PCR using expression plasmid pET15b‐*laao*/*mog* as a template and the QuikChange kit (Stratagene, La Jolla, CA, USA) using primers P1 to P10 (Table S1). For site‐directed mutagenesis, both a direct and a reverse primer were designed complementary to opposite strands of the same DNA region. The mutated genes were completely sequenced using an ABI PRISM 3500 genetic analyzer (Thermo Fisher Scientific, Waltham, MA, USA) to ensure that only the desired mutations were introduced. Each colony was cultivated in a 96‐well plate, and crude extracts were prepared using BugBuster reagent (Merck Millipore, Billerica, MA, USA).

Random mutagenesis was introduced using primers P11 and P12 (Table S1), in which target amino acid positions were replaced with a degenerate codon, NNS (N = ATGC, S = GC). Each colony was cultivated in a 96‐well plate, and crude extracts were prepared using BugBuster reagent (Merck Millipore). We screened 384 colonies (4 plates), and the library size was more than 10 times larger than the number of possible 32 cases by the NNS codon.

### Enzyme assay and protein measurement

l‐Amino acid oxidase activity was assayed by measuring the rate of hydrogen peroxide formation at pH 7.0 (for l‐Lys as a substrate), or pH 9.0 (for l‐Arg and l‐Orn as substrates). The standard reaction mixture (1.0 mL) contained 5 mm (or 0.1 mm) l‐amino acid, 0.12 mm 4‐aminoantipyrine, 0.38 mm phenol, 1.8 units of horseradish peroxidase, 0.1 m potassium phosphate buffer, pH 7.0, or 0.1 m borate buffer, pH 9.0. To investigate substrate specificity of variants, 1.0 mm each of l‐Ala, l‐Cys, l‐Asp, l‐Glu, l‐Phe, Gly, l‐His, l‐Ile, l‐Lys, l‐Leu, l‐Met, l‐Asn, l‐Pro, l‐Gln, l‐Arg, l‐Ser, l‐Thr, l‐Val, l‐Trp, l‐Tyr, d‐Lys, d‐Arg, and d‐Orn was used for substrates. The assay of enzyme activity was started by addition of enzyme solution, and formation of hydrogen peroxide was spectrophotometrically followed at 30 °C for 5 min by measuring the absorbance at 555 nm.

l‐Amino acid monooxygenase activity was assayed by measuring the rate of hydrogen peroxide formation from 5‐aminopentanamide, 4‐aminobutanamide, and 4‐guanidinobutanamide, which were monooxygenation products of l‐Lys, l‐Orn, and l‐Arg, respectively, by coupling with the purified aminoamide oxidizing enzyme from *Aspergillus carbonarius* AIU 205 29 as follows. The standard reaction mixture (1.0 mL) contained 5 mm l‐Lys, 0.12 mm 4‐aminoantipyrine, 0.38 mm *N*‐ethyl‐*N*‐(2‐hydroxy‐3‐sulfopropyl)‐3‐methylaniline, 1.8 units of horseradish peroxidase, 0.4 units of the aminoamide oxidizing enzyme, 0.1 m potassium phosphate buffer, pH 7.0, or 0.1 m borate buffer, pH 9.0. The enzyme activity assay was started by addition of enzyme solution, and the reaction was followed at 30 °C for 5 min by measuring the absorbance at 555 nm.

One unit of enzyme activity for the above three enzymes was defined as the amount of enzyme catalyzing formation of one micromole of hydrogen peroxide per min.

## Author contributions

DI, TA, and SF performed the crystallographic analysis. DM performed the mutational and enzymatic analyses. KI, YA, and SF conceived and coordinated the study. DI, DM, and SF analyzed the data and wrote the study. All authors reviewed the results and approved the final version of the manuscript.

## Supporting information


**Fig. S1.** (A) UV‐visible absorbance spectra and kinetics of l‐lysine oxidation by the wild‐type enzyme (●) and D238A (▲), D238E (♦), and D238K (■) mutants.
**Table S1**. Primers used in the present study.

## References

[1] Isobe K , Sugawara A , Domon H , Fukuta Y and Asano Y (2012) Purification and characterization of an l‐amino acid oxidase from *Pseudomonas* sp. AIU 813. J Biosci Bioeng 114, 257–261. 22704811 10.1016/j.jbiosc.2012.04.020

[2] Matsui D , Im DH , Sugawara A , Fukuta Y , Fushinobu S , Isobe K and Asano Y (2014) Mutational and crystallographic analysis of l‐amino acid oxidase/monooxygenase from *Pseudomonas* sp. AIU 813: Interconversion between oxidase and monooxygenase activities. FEBS Open Bio 4, 220–228. 10.1016/j.fob.2014.02.002 PMC3970082 24693490

[3] Du XY and Clemetson KJ (2002) Snake venom l‐amino acid oxidases. Toxicon 40, 659–665. 12175601 10.1016/s0041-0101(02)00102-2

[4] Arima J , Sasaki C , Sakaguchi C , Mizuno H , Tamura T , Kashima A , Kusakabe H , Sugio S and Inagaki K (2009) Structural characterization of l‐glutamate oxidase from *Streptomyces* sp. X‐119‐6. FEBS J 276, 3894–3903. 19531050 10.1111/j.1742-4658.2009.07103.x

[5] Ida K , Kurabayashi M , Suguro M , Hiruma Y , Hikima T , Yamomoto M and Suzuki H (2008) Structural basis of proteolytic activation of l‐phenylalanine oxidase from *Pseudomonas* sp. P‐501. J Biol Chem 283, 16584–16590. 18417467 10.1074/jbc.M800366200

[6] Ida K , Suguro M and Suzuki H (2011) High resolution X‐ray crystal structures of l‐phenylalanine oxidase (deaminating and decarboxylating) from *Pseudomonas* sp P‐501. Structures of the enzyme‐ligand complex and catalytic mechanism. J Biochem 150, 659–669. 21841183 10.1093/jb/mvr103

[7] Gaweska HM , Taylor AB , Hart PJ and Fitzpatrick PF (2013) Structure of the flavoprotein tryptophan 2‐monooxygenase, a key enzyme in the formation of galls in plants. Biochemistry 52, 2620–2626. 23521653 10.1021/bi4001563 PMC3635830

[8] Fitzpatrick PF (2010) Oxidation of amines by flavoproteins. Arch Biochem Biophys 493, 13–25. 19651103 10.1016/j.abb.2009.07.019 PMC2812625

[9] Zeldin OBGM and Garman EF (2013) RADDOSE‐3D: time‐ and space‐resolved modelling of dose in macromolecular crystallography. J Appl Crystallogr 46, 1225–1230.

[10] Moustafa IM , Foster S , Lyubimov AY and Vrielink A (2006) Crystal structure of LAAO from *Calloselasma rhodostoma* with an l‐phenylalanine substrate: insights into structure and mechanism. J Mol Biol 364, 991–1002. 17046020 10.1016/j.jmb.2006.09.032 PMC2018609

[11] Faust A , Niefind K , Hummel W and Schomburg D (2007) The structure of a bacterial l‐amino acid oxidase from *Rhodococcus opacus* gives new evidence for the hydride mechanism for dehydrogenation. J Mol Biol 367, 234–248. 17234209 10.1016/j.jmb.2006.11.071

[12] Li M , Binda C , Mattevi A and Edmondson DE (2006) Functional role of the “aromatic cage” in human monoamine oxidase B: structures and catalytic properties of Tyr435 mutant proteins. Biochemistry 45, 4775–4784. 16605246 10.1021/bi051847g

[13] Baron R , Riley C , Chenprakhon P , Thotsaporn K , Winter RT , Alfieri A , Forneris F , van Berkel WJ , Chaiyen P , Fraaije MW *et al* (2009) Multiple pathways guide oxygen diffusion into flavoenzyme active sites. Proc Natl Acad Sci USA 106, 10603–10608. 19541622 10.1073/pnas.0903809106 PMC2698890

[14] Mattevi A (2006) To be or not to be an oxidase: challenging the oxygen reactivity of flavoenzymes. Trends Biochem Sci 31, 276–283. 16600599 10.1016/j.tibs.2006.03.003

[15] Ballou DP , Entsch B and Cole LJ (2005) Dynamics involved in catalysis by single‐component and two‐component flavin‐dependent aromatic hydroxylases. Biochem Biophys Res Commun 338, 590–598. 16236251 10.1016/j.bbrc.2005.09.081

[16] Sucharitakul J , Prongjit M , Haltrich D and Chaiyen P (2008) Detection of a C4a‐hydroperoxyflavin intermediate in the reaction of a flavoprotein oxidase. Biochemistry 47, 8485–8490. 18652479 10.1021/bi801039d

[17] Okazaki S , Nakano S , Matsui D , Akaji S , Inagaki K and Asano Y (2013) X‐ray crystallographic evidence for the presence of the cysteine tryptophylquinone cofactor in l‐lysine epsilon‐oxidase from *Marinomonas mediterranea* . J Biochem 154, 233–236. 23908359 10.1093/jb/mvt070

[18] Amano M , Mizuguchi H , Sano T , Kondo H , Shinyashiki K , Inagaki J , Tamura T , Kawaguchi T , Kusakabe H , Imada K *et al* (2015) Recombinant expression, molecular characterization and crystal structure of antitumor enzyme, l‐lysine alpha‐oxidase from *Trichoderma viride* . J Biochem 157, 549–559. 25648943 10.1093/jb/mvv012

[19] Saito Y , Mifune M , Nakashima S , Odo J , Tanaka Y , Chikuma M and Tanaka H (1987) Determination of hydrogen peroxide with *N,N*‐diethylaniline and 4‐aminoantipyrine by use of an anion‐exchange resin modified with manganese‐tetrakis(sulphophenyl)porphine, as a substitute for peroxidase. Talanta 34, 667–669. 18964384 10.1016/0039-9140(87)80086-3

[20] Nakamura K , Fujii T , Kato Y , Asano Y and Cooper AJ (1996) Quantitation of l‐amino acids by substrate recycling between an aminotransferase and a dehydrogenase: application to the determination of l‐phenylalanine in human blood. Anal Biochem 234, 19–22. 8742076 10.1006/abio.1996.0043

[21] Tachibana S , Suzuki M and Asano Y (2006) Application of an enzyme chip to the microquantification of l‐phenylalanine. Anal Biochem 359, 72–78. 17046706 10.1016/j.ab.2006.09.006

[22] Yamasaki‐Yashiki S , Tachibana S and Asano Y (2012) Determination of L‐methionine using methionine‐specific dehydrogenase for diagnosis of homocystinuria due to cystathionine beta‐synthase deficiency. Anal Biochem 428, 143–149. 22750517 10.1016/j.ab.2012.06.019

[23] Otwinowski Z and Minor W (1997) Processing of X‐ray diffraction data collected in oscillation mode. Methods Enzymol 276, 307–326. 10.1016/S0076-6879(97)76066-X 27754618

[24] Kabsch W (2010) Xds. Acta Crystallogr D Biol Crystallogr 66, 125–132. 20124692 10.1107/S0907444909047337 PMC2815665

[25] Emsley P and Cowtan K (2004) Coot: model‐building tools for molecular graphics. Acta Crystallogr D Biol Crystallogr 60, 2126–2132. 15572765 10.1107/S0907444904019158

[26] Murshudov GN , Vagin AA and Dodson EJ (1997) Refinement of macromolecular structures by the maximum‐likelihood method. Acta Crystallogr D Biol Crystallogr 53, 240–255. 15299926 10.1107/S0907444996012255

[27] Adams PD , Grosse‐Kunstleve RW , Hung LW , Ioerger TR , McCoy AJ , Moriarty NW , Read RJ , Sacchettini JC , Sauter NK and Terwilliger TC (2002) PHENIX: building new software for automated crystallographic structure determination. Acta Crystallogr D Biol Crystallogr 58, 1948–1954. 12393927 10.1107/s0907444902016657

[28] Chovancova E , Pavelka A , Benes P , Strnad O , Brezovsky J , Kozlikova B , Gora A , Sustr V , Klvana M , Medek P *et al* (2012) CAVER 3.0: a tool for the analysis of transport pathways in dynamic protein structures. PLoS Comput Biol 8, e1002708. 23093919 10.1371/journal.pcbi.1002708 PMC3475669

[29] Sugawara A , Matsui D , Yamada M , Asano Y and Isobe K (2015) Characterization of two amine oxidases from *Aspergillus carbonarius* AIU 205. J Biosci Bioeng 119, 629–635. 25468423 10.1016/j.jbiosc.2014.10.023

