# Supplementary material for: Ligand complex structures of l‐amino acid oxidase/monooxygenase from Pseudomonas sp. AIU 813 and its conformational change
Source: FEBS Open Bio. 2018 Feb 8;8(3):314–24. doi: 10.1002/2211-5463.12387 (PMC5832979; doi:10.1002/2211-5463.12387)
Supplement: Supplementary file 1 — Fig. S1. (A) UV‐visible absorbance spectra and kinetics of l‐lysine oxidation by the wild‐type enzyme (●) and D238A (▲), D238E (♦), and D238K (■) mutants. Table S1. Primers used in the present study. [file FEB4-8-314-s001.pdf]

Supporting information

Table S1. Primers used in the present study

Name	Sequence	Objective
P1	5'-GGT GGT TGG GAT TCG **GCG** TTC CCC AAC TCG ATG-3'	Site-directed mutagenesis of D238A
P2	5'-CAT CGA GTT GGG GAA **CGC** CGA ATC CCA ACC ACC-3'	Site-directed mutagenesis of D238A
P3	5'-GGT GGT TGG GAT TCG **AAA** TTC CCC AAC TCG ATG-3'	Site-directed mutagenesis of D238K
P4	5'-CAT CGA GTT GGG GAA **TTT** CGA ATC CCA ACC ACC-3'	Site-directed mutagenesis of D238K
P5	5'-GGT GGT TGG GAT TCG **GAA** TTC CCC AAC TCG ATG-3'	Site-directed mutagenesis of D238E
P6	5'-CAT CGA GTT GGG GAA **TTC** CGA ATC CCA ACC ACC-3'	Site-directed mutagenesis of D238E
P7	5'-GGT GGT TGG GAT TCG **AAC** TTC CCC AAC TCG ATG-3'	Site-directed mutagenesis of D238N
P8	5'-CAT CGA GTT GGG GAA **GTT** CGA ATC CCA ACC ACC-3'	Site-directed mutagenesis of D238N
P9	5'-GGT GGT TGG GAT TCG **GTG** TTC CCC AAC TCG ATG-3'	Site-directed mutagenesis of D238V
P10	5'-CAT CGA GTT GGG GAA **CAC** CGA ATC CCA ACC ACC-3'	Site-directed mutagenesis of D238V
P11	5'-GGT GGT TGG GAT TCG **NNS** TTC CCC AAC TCG ATG-3'	Saturation mutagenesis of D238
P12	5'-CAT CGA GTT GGG GAA **SNN** CGA ATC CCA ACC ACC-3'	Saturation mutagenesis of D238

Sequences shown in bold type are mutation points.

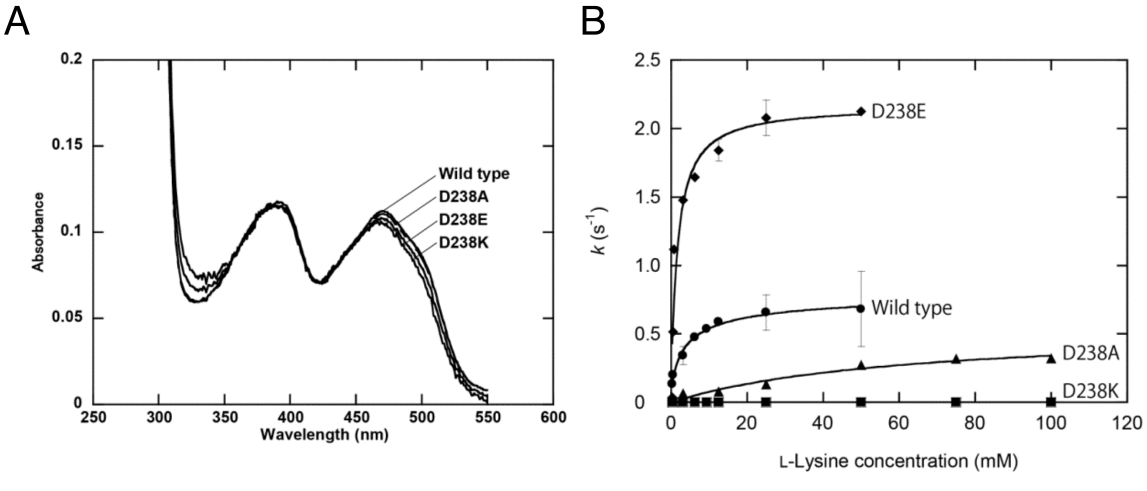

Fig. S1. (A) UV-visible absorbance spectra and kinetics of L-lysine oxidation by the wild-type enzyme (●) and D238A (▲), D238E (♦), and D238K (■) mutants.
